# Otolaryngology Care Disparities in American Indian Populations

**DOI:** 10.1002/oto2.124

**Published:** 2024-03-15

**Authors:** Alan W. Wang, Evan A. Patel, Nina Patel, Trevor A. Poulson, Ashok A. Jagasia

**Affiliations:** ^1^ Chicago Medical School Rosalind Franklin University North Chicago Illinois USA; ^2^ Department of Otolaryngology–Head and Neck Surgery Rush University Medical Center Chicago Illinois USA; ^3^ Department of Otolaryngology–Head and Neck Surgery Loyola University Stritch School of Medicine Maywood Illinois USA

**Keywords:** clinical research, health care disparities, quality of life

## Abstract

Our objectives were to quantify geographical disparities in otolaryngology care access with respect to American Indian (AI) populations and to identify gaps in care. Although increased incidence and mortality rates of ear, nose, and throat (ENT) conditions in AI populations are well documented, few studies address factors contributing to these differential outcomes. We conducted a cross‐sectional study of US states with AI areas that either met the population threshold for the American Community Survey annual estimate or annual supplemental estimate. A 2‐tailed *t* test was used to compare the geographic distribution of ENT providers practicing within AI areas against non‐AI areas, showing a statistically significant difference (*P* < .001) in the concentration of providers (0.409 vs 2.233 providers per 100,000 patients). To our knowledge, this is the first study to explore geographic barriers contributing to AI disparities within otolaryngology.

The American Indian (AI) population is an indigenous group that has long experienced large disparities in health care outcomes compared to other US populations,^1^ including within the field of otolaryngology.^2^, ^3^ Despite this, there has been minimal investigation into the driving factors behind these disparities. In addition to socioeconomic factors like higher poverty rates, another likely contributing factor is geographic isolation,^4^ especially considering that staging at presentation is the most important prognostic factor in head and neck cancer.^5^ Studies investigating survival rates of head and neck cancer noted that AI patients were more likely to present with later stages of cancer and more likely to live more than 1 hour away from a cancer center.^4^, ^5^ With geographic access likely contributing to these disparities, we seek to better quantify the distribution of ear, nose, and throat (ENT) care in AI population areas, which we hypothesize will be lower than in non‐AI areas. With a strong desire to bridge equity and access, we hope that this piece can be a first step in addressing an important gap in the literature and inspire further work.

## Methods

**Figure 1:**
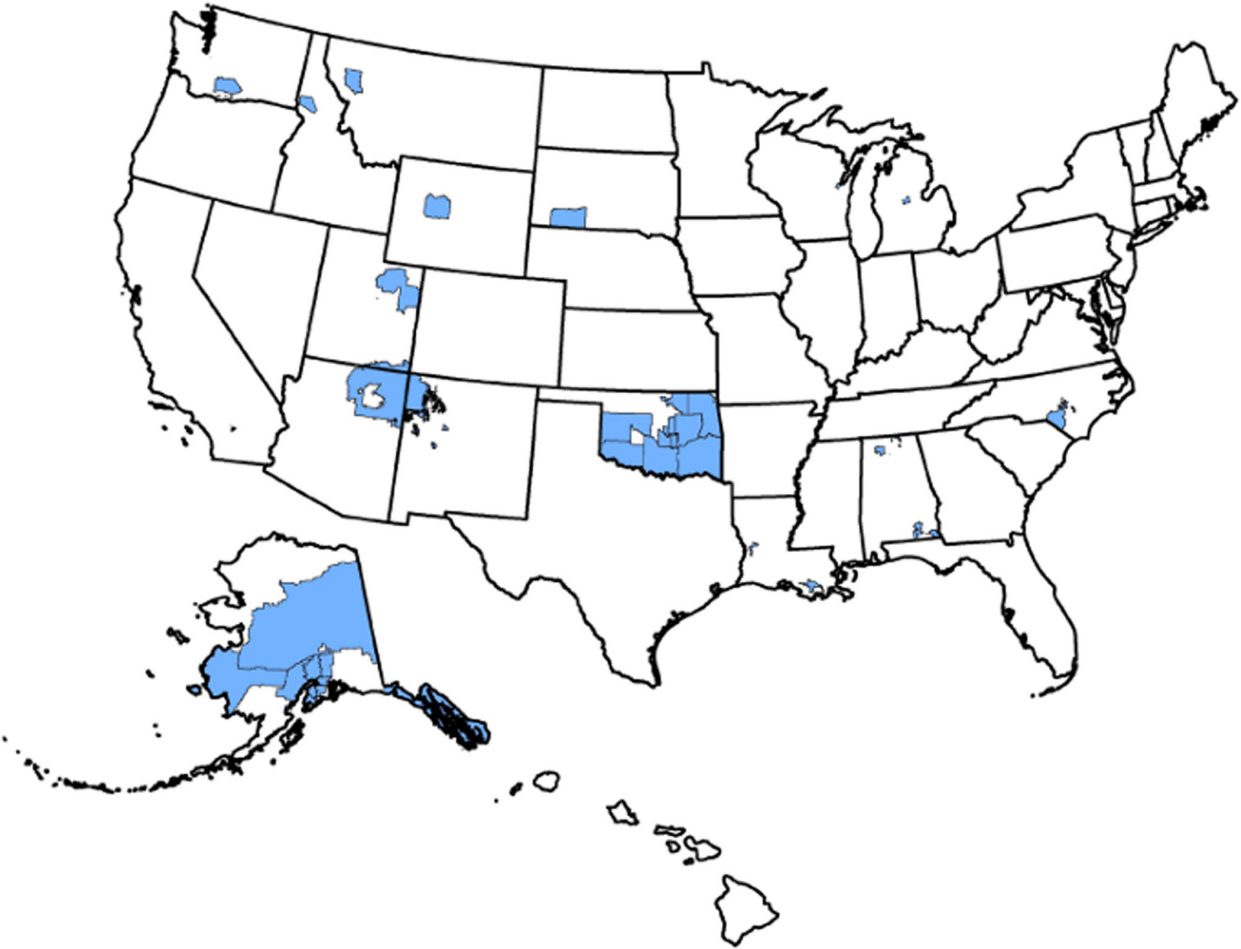
American India areas for which American Community Survey (ACS) 1‐Year estimates or ACS 1‐year supplemental estimates were released.^6^

US states selected for analysis were those with at least 1 AI area that either met the 65,000 population threshold for annual American Community Survey (ACS) estimates or the 20,000 population threshold for ACS supplemental estimates (Figure 1). The ACS is an annual survey conducted by the US Census Bureau for the purpose of collecting nationwide estimates regarding social and demographic factors.^6^ Data are also published for specific populations, including data for AI and Alaska Native (AN) areas. These AI and AN areas include: AI reservations, off‐reservation trust lands, Oklahoma tribal statistical areas, tribal designated statistical areas, state‐designated tribal statistical areas, AN regional corporations, and AN village statistical areas.^6^ Although AN populations are often grouped together with AI populations, we elected to exclude AN populations in our analysis, as all of Alaska's geographic area is encompassed by AN areas with no non‐AN areas that could be used as a comparison group. Excluding Alaska, 15 US states had AI areas that met ACS population threshold criteria.

Population metrics of AI areas were obtained using data from the 2017 to 2021 ACS via the US Census Bureau “My Tribal Area” tool, while practice locations of otolaryngologists were retrieved using the American Academy of Otolaryngology–Head and Neck Surgery (AAO‐HNS) provider database. AI areas and provider locations were mapped using publicly available datasets via ArcGIS software. Availability of otolaryngologists was expressed as the number of providers per 100,000 residents for both AI areas and non‐AI areas (Table 1), and a 2‐tailed *t* test was performed. Public information regarding geographic distance to ENT services is limited at present. A letter of exemption for this study was obtained from the investigational review board of Rush University Medical Center in Chicago, Illinois.

**Table 1:** Population of AI and Non‐AI areas and Concentration of ENT Provider

States	Population on AI land	Providers on AI land	Population on non‐AI land	Providers on non‐AI land
Alabama	194,691	1.541	4,879,605	2.664
Arizona	255,592	0	7,103,605	1.717
Utah	196,868	0	3,074,748	2.374
New Mexico	219,243	0	1,898,279	2.002
California	71,190	0	38,958,152	1.766
Idaho	33,684	0	1,905,249	1.627
Louisiana	227,416	2.638	4,362,825	3.14
Michigan	34,938	0	9,999,175	2.31
Montana	80,067	0	1,042,800	1.822
North Carolina	610,851	0.819	10,088,122	2.122
Oklahoma	2,626,357	1.142	1,393,443	1.866
South Dakota	80,518	0	829,306	2.748
Washington	171,605	0	7,614,181	2.075
Wisconsin	47,397	0	5,845,142	2.395

Abbreviations: AI, American India; ENT, ear, nose, and throat.

## Results

The mean number of otolaryngologists per 100,000 individuals in AI areas was significantly lower than the mean in non‐AI areas (0.409 vs 2.233, *P* < .001). Furthermore, 11 of the 15 selected states did not have any ENT providers within their respective AI areas. Also of note is that the average number of otolaryngologists per 100,000 individuals on non‐AI areas (mean 2.07) is nearly 1 standard deviation lower than the nationwide mean (mean 2.66, SD 0.66).^7^

These results support our hypothesis that the geographic distribution of ENT care within AI areas is lower than in non‐AI areas, and support statements made by previous studies hypothesizing that distance and geographic access to ENT care contributes to disparities experienced by AI populations.^4^, ^5^

Limitations to our study must be addressed. AI population metrics were obtained from the 2017 to 2021 ACS, which may not be representative of current data. Additionally, this data does not encompass all AI people, as a majority reside outside of AI areas.^8^ Otolaryngologist provider data was obtained from the AAO‐HNS, which only lists providers registered with the organization. The distribution of providers not registered with the AAO‐HNS may affect overall provider availability. Our analysis also only took into account the 15 states we deemed the most representative—care must be taken when generalizing findings to the rest of the US population.

## Discussion

Although preliminary in nature, our analysis highlights some of the disparities in otolaryngology care that residents of AI areas experience compared to other US populations. The low number of otolaryngologists even in non‐AI areas within these sampled states may warrant further investigation as well.

The lack of access to adequate otolaryngology care on AI land is a major concern that warrants attention from policymakers, health care providers, and the public, especially given that AI populations experience higher rates of head and neck cancer and later presentations with reduced survival.^2^, ^9^ The main health system in these regions, The Indian Health Service, aims to provide quality health care to AI populations but lacks formalized partnerships with academic medical centers, hindering the ability to recruit and retain physicians, contributing to high physician vacancy rates.^10^

We must work toward ensuring that all individuals, regardless of background or location, have access to quality health care.

This study serves to spur conversation and awareness of disparities faced by AI populations and to inspire future efforts aimed at addressing the challenges that these populations face in accessing otolaryngology care. We hope that this study will inform further research, data generation, and more thorough models for tracking geographic accessibility to otolaryngologic care for AI populations.

## Author Contributions

**Alan W. Wang**, drafted, edited, and approved the final manuscript and takes full responsibility for its content. **Evan A. Patel**, drafted, edited, and approved the final manuscript and takes full responsibility for its content. **Nina Patel**, drafted, edited, and approved the final manuscript and takes full responsibility for its content. **Trevor A. Poulson**, drafted, edited, and approved the final manuscript and takes full responsibility for its content. **Ashok A. Jagasia**, drafted, edited, and approved the final manuscript and takes full responsibility for its content.

## Disclosures

### Competing interests

None.

### Funding source

None.
